# An International Competency Framework for High-Quality Workforce Development in Integrated Care (IC): A Modified Delphi Study Among Global Participants

**DOI:** 10.5334/ijic.8258

**Published:** 2024-04-29

**Authors:** Frances Barraclough, Jennifer Smith-merry, Viktoria Stein, Sabrina Pit

**Affiliations:** 1 University Centre for Rural Health, School of Health Sciences, Faculty of Medicine and Health, The University of Sydney, NSW, Australia; 2 Centre for Disability Research and Policy, School of Health Sciences, Faculty of Medicine and Health, The University of Sydney, NSW, Australia; 3 Co-CEO VM Partners Integrating Health and Care, Department for Public Health and Primary Care, Leiden University Medical Centre, 2333, The Hague, The Netherlands; 4 Work Wiser International, Lennox Head, NSW, Australia; 5 University Centre for Rural Health, Faculty of Medicine and Health, The University of Sydney, NSW, Australia; 6 School of medicine, Western Sydney University, NSW, Australia

**Keywords:** integrated care, workforce development, competencies, Delphi study, education, training, health workforce, education framework

## Abstract

**Introduction:**

There have been increasing calls in the literature recommending training in integrated care (IC) for health and social care professionals. Although studies have focused on different stakeholders’ perceptions of education and training, there is no consistent definition of the key competencies or approach to implementing these competencies among health and social care providers. This study used a modified Delphi consensus-building method with global panellists with experience in delivering and designing training in IC to ascertain which competencies are important in an international framework guiding workforce development in IC.

**Methods:**

A four-step methodological process was used. First, a scoping review identified a potential list of competencies and features of education and training in IC. Second, predefined criteria were used to identify global panellists with IC education experience. Third, two anonymous iterative Delphi rounds were conducted to (1) reach a consensus on the level of importance of the competencies and key themes to be included and (2) identify existing models of training in IC. This was followed by the analysis of the Delphi study and presentation of the results.

**Results:**

A list of eight domains and 40 competencies was generated. Twenty-one panellists reviewed the competencies in the first and second round. The highest importance rankings were allocated to person-centred care, interprofessional teamwork and care coordination. The lower-ranking domains focused on professional workforce attributes.

**Discussion and conclusion:**

The study provides a global consensus on the competencies required for workforce training and development in IC and offers recommendations on how these competencies can be implemented in higher education and vocational institutions and workplace settings. The results will be useful for developing policy and curriculum by health and education providers and accreditation bodies.

## Introduction

A formalised structure for developing competence in integrated care (IC), such as an overarching framework outlining key competencies for a broad range of health and social care practitioners, is not well described in the literature, despite international recommendations to practise IC [1,2,3,4]. Research in IC workforce development within the last 10 years has focused on training within specific contexts (e.g., mental health, behavioural health) [5,6] and in individual disciplines, such as social work [5,6,7,8] and in undergraduate and general practice medical training [9]. This research has resulted in limited formal implementation and accreditation [6], resulting in narrowly designed curricula often developed without the input of professionals from a range of disciplines, global experts with experience in delivering training in IC and service users and providers.

There are directives from the World Health Organization (WHO) endorsing a global framework on integrated people-centred health services [10], including the WHO Global Strategy on Human Resources for Health [11] and the WHO Europe Working Document on Competencies for Integrated Care [10]. However, there appears to be no global consensus on what these training programs should look like or an agreed list of IC competencies. Although other frameworks complement IC, such as The UK Interprofessional Capability Framework [12], the Canadian Interprofessional Competency Framework [13] and the Australian Curtin University Interprofessional Capability Framework [14] none is specific to IC or developed with input from global experts in this field. Therefore, consensus on a set of core competencies relevant to health and social care providers is needed to provide standards for practice and to develop an overarching framework.

This study aims to analyse the perspectives of IC education leaders globally, within university, health and social care settings to identify what competencies a broad range of health and social care workers need to practise IC and the most effective models to implement these competencies. Meticulous, well-informed planning, development and implementation of an international competency framework will allow education providers, curriculum developers, accreditation bodies and service providers to incorporate these competencies and proposed training models into the curriculum and training programs for our health and social care workforces. The framework is relevant for health and social care professionals across a broad range of acute, community and primary care settings.

## Methods

This study used the Delphi methodology to consult panellists from various settings and countries with experience developing and delivering IC education and training. The study identified:

1. Which IC competencies are important to include in an international IC training and education framework
2. Key overarching domains
3. Recommendations of how to implement these competencies.

A Delphi study format was chosen as it allowed input from individuals with experience in workforce training in IC from various teaching institutions worldwide or authors who have published on the topic. This group was determined to have the requisite knowledge and practice-based experience to be able to determine which competencies were likely to be useful. The Delphi format was effective for identifying gaps in the evidence through input from practice and including many different perspectives in a structured way. The Delphi study method also facilitated anonymous input from panellists, avoiding control of the consensus process by individual participants [15].

We conducted a four-stage methodological process with nine evaluation points, as recommended by Nasa et al. [16]. This process strengthened the overall quality of the Delphi study by following a structured, tested approach. The four stages were:

1. Systematically identifying the problem area.
2. Selecting panel members using objective and predefined criteria.
3. Two Delphi iterative rounds, ensuring anonymity of panellists’ responses and providing controlled feedback to the panellists in between the rounds.
4. Developing the closing criteria to demonstrate consensus and stability.

**Figure 1:**
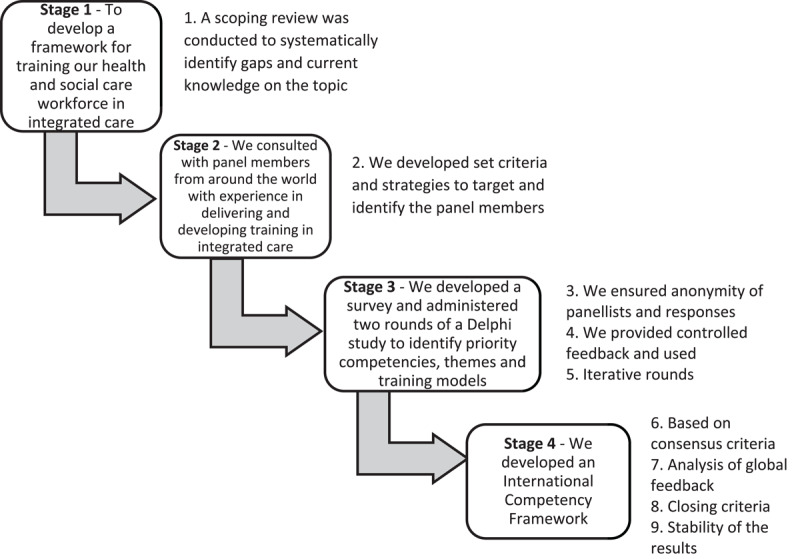
Four-stage Delphi study methodological process including nine evaluation points recommended by Nasa et al. [16].

Figure 1 outlines this process including the nine evaluation points.

The aspects of this study involving participants were approved by the Human Research Ethics Committee of the University of Sydney (HREC number 2021/701). Participants were sent details about the study; this was also attached to the REDCap survey tool; consent was indicated by participants completing the survey. Participants could withdraw from the study up to 1 month after completing the survey and ask for their data to be destroyed. All data was stored under a University of Sydney supported and licenced platform maintained by the University of Sydney. Only the University of Sydney based researchers had access to this data.

### Stage 1: Systematic identification of the problem area

To identify the problem area, we completed and published a scoping review in 2021 of the existing literature on workforce development in integrated care [17]. We searched Medline, CINAHL, EMBASE, ERIC (education, policy and theory), Cochrane, Web of Science and Scopus databases including the following search terms: integrated care, health workforce development and health workforce training. Article titles and abstracts were screened to ensure that they explicitly discussed health workforce training, education and integrated care. As part of this scoping review, we identified the barriers to implementing workforce training and education in IC and we generated a list of competencies and key themes to include in the Delphi study. The scoping review identified 33 competencies and 17 themes. These formed the basis of the questionnaire for Round 1 of the Delphi study.

### Stage 2: Selection of the panel members

Panel members were selected based on the following criteria: professional experience in designing or delivering education and training specific to IC or authors who have published on the topic. We used several approaches to identify the key panellists. First, we invited the nominated contact authors of the 62 papers from the scoping review as dedicated academic representatives. If these authors could not be contacted, an invitation was sent to subsequent authors. An open link and invitation were also sent to the International Foundation of Integrated Care’s special interest groups for education, training and research. Expressions of interest were invited through the International Foundation of Integrated Care newsletter, website and social media. Targeted invitations to participate in the Delphi study were also emailed to potential panellists personally known by the researchers as being experts in the field. Passive snowballing was used, where potential panellists were asked to pass on an invitation to participate in the study to other experts involved in IC education and training. We used this recruitment technique to engage with as many potential panellists as possible across different countries to achieve a broader perspective and consensus [15].

We aimed for a sample size of 11–30 panellists, which is typical for the Delphi method and is effective and statistically reliable [15,16]. Literature supports that having more than 30 participants does not appear to improve the quality of the results [16,21]. The generalizability of Delphi results was achieved by ensuring an appropriate panel size and diverse representation of panellists who taught different health or social care professionals in a variety of teaching organisations and countries. Panellists’ details were kept confidential.

### Survey administration

The survey included an introduction to the study providing information about the study purpose, contact details of the research team, the Delphi study process and how panellists could consent to participate.

Research Electronic Data Capture (REDCap) survey software was used to administer the questionnaires. This system provided a secure, web-based software platform designed to support real-time data collection, allowing the researchers to review the most recent results at any time [18,19].

Survey length is critical to survey fatigue and dropout rates [20]. The researchers therefore pretested the survey and determined that the average time required to complete it was 25 minutes.

### Stage 3: Delphi study rounds

Using two rounds over eight months, a modified Delphi technique was used to determine consensus on which competencies and themes from Stage 1 were important to include in an overall framework. The survey was used to structure group opinion, assess priorities and quantify the judgement of experienced educators and researchers in this field [17,18]. The process allowed panellists to contribute anonymous opinions and interpretations of the questions to reduce any likelihood of individual dominance or group conformity [19,20,21].

### Delphi Round 1

In Round 1 of the Delphi study, we asked panellists to rank the 33 competencies on a five-point Likert scale of least important (5) to most important (1). We also asked the panellists if there were any additional competencies they would like to add. We asked them to rank the 17 themes using the same process.

We included additional demographic questions about the panellists, such as gender, age, country of residence, years of experience in teaching IC and the type of organisation they work in. We then asked questions about the training they provided, including the types of training or programs, when the training was first implemented, how it was delivered, the length of the training and targeted participants. We also asked for a short description of the program.

Open questions were used to learn more about any additional competencies and themes and elicit more details about the programs. Twenty-one participants completed round 1 of the Delphi study and were invited to participate in round 2.

See Appendix A (Supplementary files), Delphi study survey Round 1.

### Delphi Round 2

At the start of the Round 2 Delphi study, panellists were given a summary of their responses from Round 1, including additional themes and competencies added by the other panellists as discussed below. See Appendix B, (Supplementary files), Delphi study survey Round 1: Results. This process is known as controlled feedback and is a classic feature of Delphi studies [20].

### Controlled feedback

The feedback included an analysis of the data from Round 1 to determine a consensus of the top-rated responses for each category. We included the average to measure central tendencies and the percent distribution for those rated “most important”. Panellists were provided with the following data about the competencies and themes:

- the number and percentage of panellists who rated each competency as “most important” (1) and the rank of each competency, in descending order.
- the average score of importance for each competency on a scale from 1 (most important) to 5 (least important).
- a summary of the analyses of additional competencies suggested.
- the number and percentage of panellists who rated each theme as “most important” (1) and the rank of each theme, in descending order.
- the average score of importance for each theme on a scale from 1 (most important) to 5 (least important).
- a summary of the analyses of additional themes suggested.

This process allowed the panellists to see trends and ascertain their position on the data presented [19,20,21]. Panellists were only asked to provide examples of their training models in Round 1. These were summarised and were used as a description in the overall framework. Panellists were not asked to rank these in Round 2; they were only asked to rank the competencies and key themes.

In between rounds, the authors including a content expert (VS) grouped the competencies under the themes to develop an overall draft framework (Framework A). This iterative process was used to obtain consensus and to continuously improve the international framework being developed [20,21].

As part of Round 2, Framework A See Appendix C (Supplementary files): Delphi study Round 2 revised framework (Framework A) was presented as an attachment so the panellists could see what the competencies and framework were beginning to look like. The panellists were then asked to rate each theme and competency individually according to how strongly they agreed they should be included in the international competency framework to build a global IC professional workforce. Panellists could also recommend changes to the wording. The authors and two other independent people piloted the survey to ensure it could be understood and how long it took to complete.

See Appendix D: (Supplementary files) Delphi study survey Round 2.

### Stage 4: Consensus criteria, closing criteria and group stability

For this study, consensus was defined as group opinion. We used the following consensus criteria [15,16]:

- if 65% of panellists agreed, the competency or theme would be kept in.
- if 30% of panellists disagreed with keeping a competency in, the competency was removed from the framework.

The closing criterion was set as two Delphi study rounds. This was acceptable due to the highly detailed scoping analyses conducted before the Delphi study. However, a third round would have been considered if group stability had not been achieved.

To achieve group stability:

1. the consensus from the scoping review of the literature informed the competencies.
2. results of Rounds 1 and 2 were compared to identify and describe what changed and what remained the same and effectively group and summarise the themes and competencies.

Two researchers (FB, SP) completed this process with shared decision-making and consultation around the results, the framework and why panellists’ recommendations on the themes and competencies were excluded, included or reworded. The repetitive and interactive survey rounds were useful in improving the wording and description of the competencies and domains and how they were listed in the overall framework.

## Results

In Round 1, 63 panellists responded wholly or partially. Only complete survey responses were included in the analysis. Round 1 was completed by 21 panellists, and 13 completed Round 2 of the survey. Reasons provided why professionals dropped out of the study included increased workload and illness during the COVID 19 pandemic.

### Descriptive characteristics of the panellists

Table 1 summarises data about the panellists who completed the survey. There was almost equal representation of males and females. Panellists were mostly from the United Kingdom (seven), followed by the Netherlands (three) and Australia (three). Nearly all the panellists (18) were working within university settings.

**Table 1:** Demographic characteristics of the Delphi study panellists (n = 21).


CHARACTERISTICS		TOTAL

Gender	Male	10

	Female	11

Country of employment	United Kingdom	7

	Netherlands	3

	Australia	3

	Austria	2

	Argentina	1

	Canada	1

	Germany	1

	Ireland	1

	Spain	1

	United States	1

Type of employment organisation*	University	18

	Hospital	4

	Other	10

Age	50–59	10

	40–49	6

	60–69	3

	30–39	2


* Multiple responses possible. (Other – TAFE, research institute, community organisation, government organisation, not for profit organisation, professional society).

### Descriptive statistics about IC training

The panellists were asked to describe the target group of participants attending their training and the mode of delivery. Most training courses identified by the participants were postgraduate programs aimed at existing healthcare workers or managers (see Table 2). These included a Master of Integrated Care, a two-year part-time online course for managers. Another master’s program was a two-year interdisciplinary Master of Population Health Management offered in the Netherlands. Table 2 summarises the characteristics of IC training.

**Table 2:** Characteristics of IC training.


CHARACTERISTICS		TOTAL

Training*	Undergraduate	4

	Postgraduate	16

	Other	5

Target participants*	Existing healthcare workers	10

	Healthcare managers	9

	Community organisations	7

Mode of delivery*	Short course (face to face)	7

	Blended	3

	Online	6


* Multiple responses possible.

### Models of training

An emphasis was placed on providing shared opportunities for interprofessional learning among disciplines and key stakeholders within the health and social care settings. The panellists emphasised the importance of integrating formalised interprofessional education into curricula early and continuing it throughout. For example, providing opportunities for health and social care workers across community and primary care settings to visit a variety of health and social care workplaces and shadow workers within these settings. This was to enhance and develop a strong mindset in relation to the delivery of IC, incorporate skills to constructively challenge others’ practice and provide regular opportunities for multidisciplinary teamwork and shared decision-making across a broader range of community and primary care settings. This would progress to supervised mentorship and care coordination with real cases after the initial training program.

Other innovative practice areas included providing opportunities for health and social care professionals to do tasks outside their traditional training to deepen their understanding of a broader range of service providers outside of the acute care setting. For example, shadowing workers from volunteer organisations and the social care sector.

The importance of including people with lived experience in all aspects of education and training was also a key feature of the panellist feedback.

Another recommendation, focusing on digital literacy, was to incorporate opportunities for health and social care professionals to communicate with people, families and other professionals and service providers through digital platforms and social media, and opportunities to learn about data as tools for design, planning and evaluation of services.

### Final framework (Framework B)

Framework B (see Table 3) presents the key domains and competencies to guide training organisations, higher education facilities, workplaces, and accreditation bodies in implementing a trained workforce in IC. The framework can guide health and social care professionals in all stages of development. The framework outlines strategies to address patient or client focused goals, organisation related goals, as well as goals centred around care coordination, digital skills and technology and practitioner attributes.

**Table 3:** Final framework (Framework B).


**Domain 1: Person-centred care**	

1.	Shared identification of the strengths and needs of individuals involving carers and families to improve quality of care, health outcomes and well-being.

2.	Developing a comprehensive understanding of individuals’ needs, including their health literacy, individual goals and how these can be met within their surrounding health and social care environment.

3.	Improving individuals’ and carers’ knowledge, skills and confidence in navigating the health and social care system.

4.	Facilitating people becoming empowered to participate in their own care, building on their strengths and capabilities.

5.	Ability to actively involve and support carers, helping them to understand and participate in care and recognising and responding to signs of carer distress.

**Organisation focused**	

**Domain 2: Interprofessional teamwork and collaborative practice**	

6.	Working effectively as a team member across a range of disciplines and settings.

7.	Collaborating with service providers within the acute, primary, and informal care sectors.

8.	Sharing information and data across teams and service providers and with individuals and their families.

9.	Demonstrating well-developed negotiation skills within teams, across services and with others in the network of care.

**Domain 3: Care coordination**	

10.	Adopting a care coordination role, including effective communication with people/carers and service providers to improve the experience of health and social care.

11.	Demonstrating the ability to coordinate care within a complex system.

12.	Demonstrating knowledge of local and national policy and programs and communicating these programs to others.

13.	The capacity to identify and collaborate with a range of professionals and key partners based on the needs of the individual, population or community.

**Domain 4: Digital skills and technology**	

14.	The ability to use a range of technology to support care coordination.

15.	Demonstrating the use of digital literacy across a broad range of settings.

16.	Engaging patients and families through technology-based communication tools to support their integrated care.

17.	Utilising linked datasets and population health management tools to analyse data and identify trends to inform and evaluate integrated care.

18.	Using shared electronic patient records to enhance communication and collaboration with service users and their families.

**Operation focused**	

**Domain 5: Health promotion and disease prevention**	

19.	Facilitating behaviour change in individuals, families and communities to achieve ways of living that promote health, resilience, well-being and disease prevention.

20.	Obtaining an integrative history that includes health and nutritional status, functional ability, housing and social circumstances, wellness strategies and use of conventional medicines and complementary therapies.

21.	Knowledge of local community resources and preventative programs to support people and communities to make healthy choices.

22.	Providing education on self-care strategies for maintaining good health and incorporate these strategies and resources into all care plans.

**Domain 6: Population health approach to care**	

23.	Identifying and addressing the needs of local communities by understanding the available resources, population data, and gaps that may exist in healthcare delivery.

24.	Demonstrating knowledge of local, national and international population health strategies and programs, and understanding when and how to access these services to support health and wellness.

25.	Understanding how to navigate system complexity across population health programs and service providers to enable integrated care.

26.	Identifying and referring vulnerable populations and those experiencing health inequalities to appropriate support programs, to plan and deliver effective care at a population level.

27.	Understanding the social determinants of health such as housing and employment and how these impact population health outcomes to support integrated care.

**Practitioner attributes**	

**Domain 7: Leadership**	

28.	Developing as leaders, role models and local champions to advocate for integrated governance and support implementation of integrated care.

29.	Demonstrating leadership in influencing other professionals and service providers to be more person-centred and collaborative in their practice.

30.	Facilitating opportunities for shared learning and innovation across disciplines, providers and partners to encourage reform and new ways of working.

31.	Creating a safe space to constructively challenge the practice of others to ensure the delivery of person-centred care.

32.	Implementing shared governance between multi-stakeholders and sectors.

33.	Enabling and creating opportunities for systems thinking and change together with service providers and people with lived experience to improve healthcare delivery and outcomes.

34.	Using evaluation and research methods including lived experience to drive change and improve services.

**Domain 8: Professional and ethical attributes**	

Members of our health and social care workforce need to be seen as role models. They need to demonstrate the following professional and ethical practitioner attributes:	

35.	practising and integrating self-care strategies

36.	engaging in continuous learning, supervision and maintaining evidence-informed practice

37.	becoming mentors, teachers and peer learners

38.	showing empathy and emotional intelligence

39.	practising reflective thinking and learning

40.	demonstrating competencies in working with difference (cultural, social and neurodiversity).


### The domains

This study identified eight overarching domains or pillars. These are person centred care, interprofessional teamwork and collaborative practice, care coordination, digital skills and technology, health promotion and disease prevention, a population health approach to care, leadership and professional and ethical attributes. Panellists recommended an additional domain to reflect the need for digital skills and the use of technology. Panellists also recommended additional competencies under this domain. The order of the domains was changed to prioritise person-centred care. The competencies were then listed under each of the domains. The initial wording of “themes” to “domains” to reflect the language of other national and international frameworks, for example the Australian Medical Council Framework for Prevocational Medical Training [22] and Capability Framework on Digital Health in Medicine [23].

### The competencies

The final framework recommends 40 competencies. The competencies focus on a broad range of skills to deliver integrated person-centred care. New competencies were recommended by the panellists during Round 1 recommending the ability to challenge the practice of others, a focus on digital literacy, and practitioner attributes including empathy, emotional intelligence, reflective thinking and explicit values such as systems thinking and leadership. New competencies added following Round 2 were based on demonstrating the ability to work with difference (cultural, social and neurodiversity).

According to the panellists consulted in the Delphi study Round 1, the highest-ranked competency elements directly related to interprofessional teamwork (ranked 1) and having a more in-depth understanding of patients: “people’s needs and how they can be met with their surrounding health and social care systems” (ranked 2 and 3). Competencies that ranked lowest related to practitioner qualities, such as practising self-care and competencies related to obtaining an integrative health history that includes mind-body-spirit, and the use of both conventional and integrative therapies.

Round 2 of the Delphi study included recommendations from Round 1 and a more refined standard of practice and framework for designing curricula and workplace continuing education programs aimed at healthcare professionals. Panellists from Round 1 were asked to look at the themes and related competencies and comment if any should be removed or extra competencies included, suggest any rewording or comment further on how the framework could be improved. Following Round 2, an additional nine competencies were added. These related to digital skills and technology and cultural, social and neurodiversity.

## Discussion

This article presents the results of a consensus-building study to identify key competencies and training models for health and social care workers in different international higher education settings. We created the framework by examining the available international literature and identifying competencies and key themes [17]. We then identified key global experts with experience and knowledge about training in integrated care to rank the competencies and build and finalise the framework. We identified eight domains and 40 competencies. The result is an overarching conceptual framework listing domains and competencies relevant to all health and social care workers which can be implemented in university, education or workplace settings. We now recommend that these are trialled in different higher education settings internationally, including undergraduate and postgraduate programs and within programs for new and existing health and social care workers and managers.

The framework builds on existing literature and reinforces the need for interprofessional learning [12,13,14] and person centredness [24,25]. What our framework adds is a guide on how to best implement these competencies to ensure people and patients are provided with personalised care centred around their goals and context, including early intervention and prevention. In addition, we consider support for carers and prioritise care beyond the acute care setting [26,27]. The focus is on collaborating with a broad range of health and social care providers. To enable this our framework recommends that we train both our health and social care providers together and that we involve people and carers with lived experience in all aspects of education and training, including design, delivery, implementation, assessment and evaluation.

The focus of our framework is on upskilling practitioners who work in health and social care as well as providing organisations and training environments with the skills necessary to deliver integrated person-centred care. The competencies are divided into person focused, organisation focused, operation focused and practitioner attributes as criteria to guide accreditation bodies, staff recruitment, developing position descriptions and managing staff performance. Our framework describes in detail the competencies and methods to achieve this including training health and social care professionals together to work in interdisciplinary teams; teaching and role modelling communication, collaboration and shared decision-making skills; care coordination; and training in providing a comprehensive assessment of patient and family needs [31,32,33]. These principles do not appear to be fully integrated into the current training and curricula for our health and social care workforce where workplaces and training organisations have limited understanding of integrated care and where health care professionals are still trained in silos [6,17,34,35]. Other key features of the curricula include a focus on digital health, and regular interprofessional learning among stakeholders in both health and social care settings, including teamwork and shared decision-making within these settings provided by supervised mentorship and shadowing opportunities.

It is possible to upskill our current and future health and social care workforce in IC with an overall framework that includes advanced competencies for managing patients with complex needs and a mutual understanding of the competencies required to practise IC [3,6]. Our framework builds on the work of the World Health Organisation framework on integrated people centred health services [24] and the whole-systems approach for person centred care within the European person-centred healthcare curriculum framework [4] by providing an education framework to enable these strategies.

Our framework can be used to guide critical discussions about curriculum content and the systems needed to implement integrated care within a variety of contexts including higher education, other learning jurisdictions and amongst health and social care providers. Higher education institutions, accreditation bodies and workplaces across the health and social care sectors are encouraged to implement and be benchmarked against the framework’s competencies to achieve IC certification. The framework can be used to guide curricula development for undergraduate and postgraduate health care professional training and postgraduate-specific programs on IC. Our framework can also be used as criteria for workplace training, staff recruitment, orientation, developing position descriptions and managing staff performance in the workplace [28,29,30].

Further work is needed to refine and test the competencies to ensure feasibility and usefulness and ensure that curriculum developers and workplaces are appropriately supported and trained to incorporate and implement the competencies. Further work also needs to focus on upskilling leaders in this area supporting innovation in how programs are delivered and utilising this framework to inform all stages of curriculum development.

The next stage of the overall study will involve interviews with university curriculum leads to see how the framework and competencies can implemented into the training of our health care workforce. Anticipated barriers include that we are still training our health professionals in silos, there is a lack of understanding of what integrated care is and how it is applied in practice, we are still training our health workforce with little or no cross over and understanding of the social care sector, we are not always including patients and carers in decision making, we are still focussing on disease based and acute focused care. Our health care workforce is not trained to coordinate care across sectors. Another barrier is that we are still focussing on interprofessional frameworks that do not include key concepts in integrated care such as care coordination across sectors and the implementation into our practice of health promotion and disease prevention. The framework can also be used to guide policy decisions and the review and implementation of accreditation standards for health and social care workers, ensuring patients and carers are part of this process.

### Limitations and strengths

The study has several strengths and limitations. First, the study was conducted during the Covid-19 pandemic. This led to challenges in the time it took to recruit panellists to complete the two stages of the Delphi study because of well documented practitioner fatigue and survey fatigue during this period [36,37]. Nearly all the panellists (n = 18) were working within university settings. This was expected, given that the curricula are developed by university staff. A strength of the study was the contribution of panellists with advanced knowledge and experience in teaching and developing IC programs in various settings.

Another key strength of this study was the iterative design. The study began with an international scoping review of the literature identifying key themes and competencies. Our study followed a rigorous process to increase the reliability and validity of the results. We followed a four-step methodological process with nine evaluation points, as recommended by Nasa et al. [16].

## Conclusions

Existing competencies and models of training to build capacity for IC are not comprehensive, and none has been developed through a formal global expert consensus technique. Higher education and other education providers, workplaces and accreditation bodies may use the results of our study to drive reform and to guide discussion about curriculum content and the systems needed to implement integrated care. The competencies can be used to shape job descriptions, orientation programs, supervision skills and performance reviews and as a resource for educators to shape existing IC curricula and training programs.

## Additional files

The additional files for this article can be found as follows:

10.5334/ijic.8258.s1 Appendix A. Delphi study survey Round 1.

10.5334/ijic.8258.s2 Appendix B. Delphi study survey Round 1: Results.

10.5334/ijic.8258.s3 Appendix C. Delphi study Round 2 revised framework (Framework A).

10.5334/ijic.8258.s4 Appendix D. Delphi study survey Round 2.

## Reviewers

**PJ Harnett**, Head of Integrated Care, Mowlam Healthcare and Adjunct Associate Professor, Trinity College Dublin, Ireland

**Débora Koatz**, Msc, Avedis Donabedian Research Institute. Universitat Autònoma de Barcelona, Barcelona, Spain *and* Chronicity, Primary Care and Health Promotion Research Network (RICAPPS-RICORS), Spain

**Deb Letica**, Citizen Leader, FRACP (Hon), Australia

**Dr. Dominika Lisiecka**, Department of Nursing and Healthcare Sciences, Munster Technological University – Kerry Campus, Tralee, Co. Kerry. Ireland

## References

[1] Amelung VE, Stein V, Goodwin N, Balicer R, Nolte E, Suter E. Handbook of integrated care. Switzerland: Springer; 2017. DOI: 10.1007/978-3-319-56103-5

[2] Mitchell G, Burridge L, Zhang J, Donald M, Scott I, Dart J, et al. Systematic review of integrated models of health care delivered at the primary–secondary interface: How effective is it and what determines effectiveness? Australian Journal of Primary Health. 2015; 21: 391–408. DOI: 10.1071/PY14172 26329878

[3] Valentijn PP, Schepman SM, Opheij W, Bruijnzeels MA. Understanding integrated care: A comprehensive conceptual framework based on the integrative functions of primary care. International Journal of Integrated Care. 2013; 13(1). DOI: 10.5334/ijic.886 PMC3653278 23687482

[4] World Health Organization. Regional Office for Europe & Health Services Delivery Programme, Division of Health Systems and Public Health. Roadmap: Strengthening people-centred health systems in the WHO European region: A framework for action towards coordinated/integrated health services delivery (CIHSD). Copenhagen: WHO Regional Office for Europe; 2013. Available from: http://www.euro.who.int/__data/assets/pdf_file/0005/231692/e96929-replacementCIHSD-Roadmap-171014b.pdf?ua=1.

[5] Yamada AM, Wenzel SL, DeBonis JA, Fenwick KM, Holguin M. Experiences of collaborative behavioral healthcare professionals: Implications for social work education and training. Journal of Social Work Education. 2019; 55(3): 519–36. DOI: 10.1080/10437797.2019.1593900

[6] Rubin M, Kilgore RC. Integrated care workforce development: University–community collaboration. Journal of Social Work Education. 2019; 39(4): 534–51. DOI: 10.1080/02615479.2019.1661987

[7] Reno R, Beaujolais B, Davis TS. Facilitating mechanisms for integrating care to promote health equity across the life course: Reflections from social work trainees. Social Work in Health Care. 2019; 58(1): 60–74. DOI: 10.1080/00981389.2018.1531105 30332345

[8] Davis TS, Reno R, Guada J, Swenson S, Peck A, Saunders-Adams S, et al. Social Worker Integrated Care Competencies Scale (SWICCS): Assessing social worker clinical competencies for health care settings. Social Work in Health Care. 2019; 58(1): 75–92. DOI: 10.1080/00981389.2018.1547346 30457040

[9] Dale J, Russell R, Harkness F, Wilkie V, Aiello M. Extended training to prepare GPs for future workforce needs. British Journal of General Practice. 2017; 67(662): E659–67. DOI: 10.3399/bjgp17X691853 PMC5569746 28716998

[10] World Health Organization. Regional Office for Europe & Health Services Delivery Programme, Division of Health Systems and Public Health. Roadmap: Strengthening people-centred health systems in the WHO European region: A framework for action towards coordinated/integrated health services delivery (CIHSD). Copenhagen: WHO Regional Office for Europe; 2013. Available from: http://www.euro.who.int/__data/assets/pdf_file/0005/231692/e96929-replacementCIHSD-Roadmap-171014b.pdf?ua=1.

[11] World Health Organization. Global strategy on human resources for health: Workforce 2030. Geneva: World Health Organization; 2016. Available from: https://apps.who.int/iris/bitstream/handle/10665/250368/9789241511131-eng.pdf.

[12] Interprofessional Education Team. Interprofessional Capability Framework 2010: Mini-Guide. http://www.health.heacademy.ac.uk/doc/resources/icf2010.pdf/at_download/file.pdf. Accessed February 25, 2014

[13] Canadian Interprofessional Health Collaborative. A National Interprofessional Competency Framework. 2010. www.cihc.ca/files/CIHC_IPCompetencies_Feb1210.pdf. Accessed February 17, 2014.

[14] Curtin Interprofessional Capability Framework. http://healthsciences.curtin.edu.au/local/docs/IP_Capability_Framework_booklet.pdf. Accessed February 17, 2014

[15] Boulkedid R, Abdoul H, Loustau M, Sibony O, Alberti C. Using and reporting the Delphi method for selecting healthcare quality indicators: A systematic review. PLOS One. 2011; 6(6): e20476. DOI: 10.1371/journal.pone.0020476 21694759 PMC3111406

[16] Nasa P, Jain R, Juneja D. Delphi methodology in healthcare research: How to decide its appropriateness. World Journal of Methodology. 2021 Jul 20; 11(4): 116–29. DOI: 10.5662/wjm.v11.i4.116 34322364 PMC8299905

[17] Barraclough F, Smith-Merry J, Stein V, Pit S. Workforce Development in Integrated Care: A Scoping Review. International Journal of Integrated Care. 2021; 21(4): 23. DOI: 10.5334/ijic.6004 PMC8622255 34899102

[18] Harris PA, Taylor R, Thielke R, Payne J, Gonzalez N, Conde JG. Research electronic data capture (REDCap): A metadata-driven methodology and workflow process for providing translational research informatics support. Journal of Biomedical Informatics. 2009 Apr; 42(2): 377–81. DOI: 10.1016/j.jbi.2008.08.010 18929686 PMC2700030

[19] Harris PA, Taylor R, Minor BL, Elliott V, Fernandez M, O’Neal L, et al. The REDCap consortium: Building an international community of software partners. Journal of Biomedical Informatics. 2019 Jul; 95: 103206. DOI: 10.1016/j.jbi.2019.103208 PMC7254481 31078660

[20] Dalkey NC. The Delphi method: An experimental study of group opinion. Santa Monica, CA: RAND Corporation; 1969. DOI: 10.1016/S0016-3287(69)80025-X

[21] Akins RB, Tolson H, Cole BR. Stability of response characteristics of a Delphi panel: Application of bootstrap data expansion. BMC Medical Research Methodology. 2005; 5: 37. DOI: 10.1186/1471-2288-5-37 16321161 PMC1318466

[22] The Australian Medical Council. (2023). National Framework for Prevocational Medical Training. https://www.amc.org.au/accredited-organisations/prevocational-training/new-national-framework-for-prevocational-pgy1-and-pgy2-medical-training-2024/.

[23] The Australian Medical Council & the Australian Digital Health Agency Digital Health in Medicine Capability Framework. (2021). https://www.amc.org.au/wp-content/uploads/2021/10/Digital-Health-in-Medicine-Capability-Framework-FINAL-18-Oct-2021.pdf.

[24] World Health Organization. (2015). WHO global strategy on people-centred and integrated health services: interim report. World Health Organization. https://apps.who.int/iris/handle/10665/155002.

[25] McCormack, B. and Dewing, J. (2019) International Community of Practice for Person-centred Practice: position statement on person-centredness in health and social care. International Practice Development Journal. 9(1): Article 3. 1–7. DOI: 10.19043/ipdj.91.003

[26] Kuipers P, Ehrlich C, Brownie S. Responding to health care complexity: Suggestions for integrated and interprofessional workplace learning. J Interprof Care. 2014; 28(3): 246–8. DOI: 10.3109/13561820.2013.821601 23914938

[27] Leijten FRM, Struckman V, van Ginnekan E, Czypionka T, Kraus M, Reiss, M, et al. The SELFIE framework for integrated care for multi-morbidity: Development and description. Health Policy. 2018; 122(1): 12–22. DOI: 10.1016/j.healthpol.2017.06.002 28668222

[28] Hoge MA, Morris JA, Laraia M, Pomerantz A, Farley T. (2014). Core competencies for integrated behavioral health and primary care. Washington, DC: SAMHSA–HRSA Center for Integrated Health Solutions.

[29] Rosenberg T, Mullin D. Building the plane in the air … but also before and after it takes flight: Considerations for training and workforce preparedness in integrated behavioural health. Int Rev Psychiatry. 2018; 30(6): 199–209. DOI: 10.1080/09540261.2019.1566117 30862259

[30] Langins M, Borgermans L. Strengthening a competent health workforce for the provision of coordinated/integrated health services. Int J Integr Care. 2016; 16(6): 1–2. DOI: 10.5334/ijic.2779

[31] Shalev D, Docherty M, Spaeth-Rublee B, Khauli N, Cheung S, Levenson J, et al. Bridging the behavioral health gap in serious illness care: Challenges and strategies for workforce development. Am J Geriatr Psychiatry. 2020; 28(4): 448–62. DOI: 10.1016/j.jagp.2019.09.003 31611044

[32] Busetto L, Calciolari S, González Ortiz LG, Luijkx K, Vrijhoef B. Integrated care and the health workforce. In Amelung V, Stein V, Goodwin N, Balicer R, Nolte E, Suter E (Eds.), Handbook integrated care. Cham, Switzerland: Springer; 2017. pp. 209–20. DOI: 10.1007/978-3-319-56103-5_13

[33] Nummela O, Juujärvi S, Sinervo T. Competence needs of integrated care in the transition of health care and social services in Finland. Int J Care Coord. 2019; 22(1): 36–45. DOI: 10.1177/2053434519828302

[34] Aiello M, Mellor JD. Integrating health and care in the 21st century workforce. J Integr Care. 2019; 27(2): 100–10. DOI: 10.1108/JICA-09-2018-0061

[35] Choi RJ, Betancourt RM, DeMarco MP, Bream KDW. Medical student exposure to integrated behavioral health. Acad Psychiatry. 2019; 43(2): 191–5. DOI: 10.1007/s40596-018-0936-0 29790101

[36] de Koning R, Egiz A, Kotecha J, Ciuculete AC, Ooi SZY, Bankole NDA, Erhabor J, Higginbotham G, Khan M, Dalle DU, Sichimba D, Bandyopadhyay S and Kanmounye US. Survey Fatigue During the COVID-19 Pandemic: An Analysis of Neurosurgery Survey Response Rates. Front. Surg. 2021; 8: 690680. DOI: 10.3389/fsurg.2021.690680 34458314 PMC8388838

[37] Rodriguez-Blazquez C, Romay-Barja M, Falcon M, Ayala A, Forjaz MJ. Psychometric Properties of the COVID-19 Pandemic Fatigue Scale: Cross-sectional Online Survey Study. JMIR Public Health Surveill. 2022; 8(9): e3467. DOI: 10.2196/34675 PMC9501671 35785547

