# Supplementary material for: An International Competency Framework for High-Quality Workforce Development in Integrated Care (IC): A Modified Delphi Study Among Global Participants
Source: Int J Integr Care. 2024 Apr 29;24(2):11. doi: 10.5334/ijic.8258 (PMC11067980; doi:10.5334/ijic.8258)
Supplement: Appendix A. — Delphi study survey Round 1. [file ijic-24-2-8258-s1.pdf]

Workforce Training in Integrated Care Delphi Study

A focus on education and training in integrated care is considered a priority for our future and current healthcare workforce. The purpose of this modified Delphi study is to identify and validate key competencies, themes and training models in integrated care ranked in order of highest importance. This study may assist curriculum developers and health workforce planners to incorporate these competencies into future curriculums and workforce training and development.

We are conducting a research study about integrated care workforce development. The purpose of this study is to analyse the interactions between integrated care education experts globally to explore what competencies are needed to practice integrated care and to identify best practice models to implement these competencies.

If you decide to take part in this study, you will be asked to complete two separate questionnaires. Each questionnaire will take approximately 20mins to complete.

Please download a copy of the participant Information Sheet for your Records

[Attachment: "USYD Participant Information Sheet (Delphi Study).pdf"]

If you would like to participate in an interview or to find out more about the project please email frances.barraclough@sydney.edu.au

You can download a copy of the interview participant information sheet here

[Attachment: "USYD Participant Information Sheet (Interviews).pdf"]

Please provide your name, remember that this will remain confidential

____

What is your current age

- ◯ 20-29 years
- ◯ 30-39 years
- ◯ 40-49 years
- ◯ 50-59 years
- ◯ 60-69 years
- ◯ 70+ years

Which gender best describes you?

- ◯ Male
- ◯ Female
- ◯ Other
- ◯ Prefer not to say

What country are you providing education and training in integrated care?

Please describe	__________________________________
What type of education programs do you deliver that relate to integrated care?	☐ University undergraduate unit of study<br>☐ University postgraduate unit of study<br>☐ Short course<br>☐ Other
Please specify	__________________________________
What year did the undergraduate program begin?	__________________________________
Mode of delivery, how is the undergraduate program delivered?	☐ Face to face<br>☐ Blended<br>☐ Online<br>☐ Practicum placement<br>☐ Individual assignments<br>☐ Group assignments
What is the length of the undergraduate program?	○ 1 week or less<br>○ 1 month or less<br>○ 1 month to 6 months<br>○ 6 months to 1 year<br>○ 1 year<br>○ 1-2 years<br>○ 2 years +
Who are your target participants for the undergraduate program?	☐ Health care students<br>☐ Existing health care workers<br>☐ Health care managers<br>☐ Participants from community organisations and other sectors<br>☐ Other
Please specify	__________________________________
Please provide a short description of the undergraduate program, include aim, objectives, content and structure and web link and targeted disciplines	____
What year did the postgraduate program begin?	__________________________________
Mode of delivery, how is the postgraduate program delivered?	☐ Face to face<br>☐ Blended<br>☐ Online<br>☐ Practicum placement<br>☐ Individual assignments<br>☐ Group assignments

What is the length of the postgraduate program?	◯ 1 week or less<br>◯ 1 month or less<br>◯ 1 month to 6 months<br>◯ 6 months to 1 year<br>◯ 1 year<br>◯ 1-2 years<br>◯ 2 years +
Who are your target participants for the postgraduate program?	☐ Health care students<br>☐ Existing health care workers<br>☐ Health care managers<br>☐ Participants from community organisations and other sectors<br>☐ Other
Please specify	__________________________________
Please provide a short description of the postgraduate program, include aim, objectives, content and structure and web link and targeted disciplines	____
What year did the short course begin?	__________________________________
Mode of delivery, how is the short course delivered?	☐ Face to face<br>☐ Blended<br>☐ Online<br>☐ Practicum placement<br>☐ Individual assignments<br>☐ Group assignments
What is the length of the short course?	◯ 1 week or less<br>◯ 1 month or less<br>◯ 1 month to 6 months<br>◯ 6 months to 1 year<br>◯ 1 year<br>◯ 1-2 years<br>◯ 2 years +
Who are your target participants for the short course?	☐ Health care students<br>☐ Existing health care workers<br>☐ Health care managers<br>☐ Participants from community organisations and other sectors<br>☐ Other
Please specify	__________________________________
Please provide a short description of the short course, include aim, objectives, content and structure and web link and targeted disciplines	____
What year did the [program_other] begin?	__________________________________

Mode of delivery, how is the [program_other] delivered?	☐ Face to face<br>☐ Blended<br>☐ Online<br>☐ Practicum placement<br>☐ Individual assignments<br>☐ Group assignments
What is the length of the [program_other]?	◯ 1 week or less<br>◯ 1 month or less<br>◯ 1 month to 6 months<br>◯ 6 months to 1 year<br>◯ 1 year<br>◯ 1-2 years<br>◯ 2 years +
Who are your target participants for the [program_other]?	☐ Health care students<br>☐ Existing health care workers<br>☐ Health care managers<br>☐ Participants from community organisations and other sectors<br>☐ Other
Please specify	__________________________________
Please provide a short description of the [program_other], include aim, objectives, content and structure and web link and targeted disciplines	____

Please rate the following competencies as they relate to integrated care. These competencies have been derived from a recent scoping review.

	1 most important	2	3	4	5 least important
Enhance workforce understanding of and exposure to alignment of activities across both the health and social care systems	◯	◯	◯	◯	◯
Enable workforce attitudes to proactively pursue depth to understand system complexity including knowledge of as well as how to access and refer to services	◯	◯	◯	◯	◯
Skills to construct a comprehensive understanding of individual patients' complex needs and how these can be met within their surrounding health and social care systems	◯	◯	◯	◯	◯
An understanding of how social and cultural factors affect health	◯	◯	◯	◯	◯
Consideration for concerns specific to vulnerable populations and their needs	◯	◯	◯	◯	◯
Skills to actively pursue depth and continuously asking 'why' (rather than just 'what' or 'how') to construct a deep understanding of individual patients (their perceptions, beliefs and psychosocial context) and the system within which they interact	◯	◯	◯	◯	◯
A holistic understanding of individuals' health and wellbeing, capabilities, self-management abilities, needs, preferences and the environment in which they find themselves, including recognition that an individual's situation is dynamic, not static and requires regular monitoring	◯	◯	◯	◯	◯

Skills to establish a longitudinal alliance with patients and their families	○	○	○	○	○
Extensive integrated knowledge of biopsychosocial aspects of disease, systems of care and social determinants of care	○	○	○	○	○
Understanding how to apply knowledge of the major determinants of health given resources available, relevant health policies and system design within a community	○	○	○	○	○
Involvement of and communication with caregivers	○	○	○	○	○
An active approach to caregiver wellness and support, including understanding risk factors, recognizing signs of caregiver distress, assessing caregiver needs and referring caregivers to care	○	○	○	○	○
Familiarity with local and national resources to support social needs and can connect patients and caregivers to such resources, including community-based partners	○	○	○	○	○
Skills to collaborate with community-based partners to improve patient care, including services outside of traditional health related settings	○	○	○	○	○
Health promotion and disease prevention, including knowledge of and referral to preventative facilities and local programmes and support for lifestyle interventions	○	○	○	○	○
Embrace individuals, communities and services as partners in care	○	○	○	○	○

A person-focused approach that considers the patient's presenting problem and other medical issues	○	○	○	○	○
Focuses on the needs of individuals, families and communities to improve their quality of care, health outcomes and wellbeing	○	○	○	○	○
Support patients in their involvement in their care by empowering them with knowledge and skills per their capabilities	○	○	○	○	○
Patient-centred and relationship-centred care	○	○	○	○	○
Work effectively as a member of an interprofessional team	○	○	○	○	○
Collaborate with individuals and families to develop a personalised care plan to promote health and wellbeing that incorporates integrative approaches, including lifestyle counselling and mind-body strategies	○	○	○	○	○
Facilitate behaviour change in individuals, families and communities to achieve ways of living that promote health, resilience, wellbeing and disease prevention	○	○	○	○	○
Obtain an integrative health history that includes mind-body-spirit, nutrition and use of both conventional and integrative therapies	○	○	○	○	○
Practice self-care	○	○	○	○	○
Demonstrate basic knowledge of the major health professions, both integrative and conventional	○	○	○	○	○
Demonstrate skills to incorporate integrative healthcare into community settings and the healthcare system at large	○	○	○	○	○

Value continuous learning, become mentors, teachers and peer learners	○	○	○	○	○
Patient centredness; understanding and facilitating patients' pathways through the care system	○	○	○	○	○
Collaborating with other providers; strong communication and collaboration skills and the ability to develop strong working relationships with team members are imperative	○	○	○	○	○
Community-based health education, health promotion and disease prevention	○	○	○	○	○
Knowledge of how to teach patients self-care strategies to stay healthy and how to incorporate the patient's strengths and resources within their care plan	○	○	○	○	○
Understanding individuals' roles in the integrated healthcare team and the ability to articulate this role to other team members	○	○	○	○	○

Can you suggest any other competencies that may be missing from this list?

____

Themes - these key themes have been derived from a recent scoping review

	1 most important	2	3	4	5 least important
Deeper understanding of our health and social care systems	○	○	○	○	○
Implementing shared learning and innovation to encourage reform thinking and new ways of working	○	○	○	○	○
Clinician/consumer collaboration: Adopting collaborative practices with consumers, carers and families and service providers working together	○	○	○	○	○
Deeper understanding of our patients	○	○	○	○	○
Deeper understanding of our communities	○	○	○	○	○
Enhanced understanding of systems and available resources	○	○	○	○	○
Caregiver involvement	○	○	○	○	○
Illness prevention	○	○	○	○	○
Interprofessional teamwork and collaborative practice	○	○	○	○	○
Empowering patients and communities	○	○	○	○	○

	1 most important	2	3	4	5 least important
Developing leaders, role models and local champions to support and implement reform and change	○	○	○	○	○
The development of skills to support person centred care	○	○	○	○	○
Skills to support people and communities to manage health and wellness	○	○	○	○	○
Health promotion and disease prevention	○	○	○	○	○
quality_of Improved quality of life for health and social care providers	○	○	○	○	○
Improved patient experience of health care	○	○	○	○	○
Improved services matched to community need	○	○	○	○	○

Can you suggest any other themes that may be missing from this list?

____

Models of Training - how do you incorporate these key competencies?

	1 most important	2	3	4	5 least important
Develop new competencies for our existing health and social care workforce to target integrated care	○	○	○	○	○
Incorporate integrated care concepts throughout the curriculum of health care providers so they become fundamental to delivering care	○	○	○	○	○
Create a working environment that values wellness and work-life balance	○	○	○	○	○
Provide trainees with education around maintaining wellness and work-life balance	○	○	○	○	○
Embed structures to support collaboration and learning across services, strengthening multisector relationships and multi-organisation training	○	○	○	○	○
Incorporate simulation-based scenarios using actors from the local community with lived experiences	○	○	○	○	○
Incorporate the need to and how to provide education and support for caregivers, including illness prevention and improving quality of life.	○	○	○	○	○

	1 most important	2	3	4	5 least important
Allow more time for networking and opportunities for individual service presentations and diverse attendance, including the social care and voluntary sectors	○	○	○	○	○
	1 most important	2	3	4	5 least important
Case studies, exercises and simulations are encouraged to allow students to interact with the content in as realistic a venue as possible	○	○	○	○	○
	1 most important	2	3	4	5 least important
Focus on soft skills, such as communication, teamwork and relationship building	○	○	○	○	○
	1 most important	2	3	4	5 least important
Focus on skills to build durable relationships with patients, other professionals, other services and caregivers	○	○	○	○	○
	1 most important	2	3	4	5 least important
Focus on self-management promotion and skills, including the use of motivational interviewing techniques	○	○	○	○	○
	1 most important	2	3	4	5 least important
Skills to navigate the health and social care systems and work on individualised care plans and assessments	○	○	○	○	○
	1 most important	2	3	4	5 least important
Mentorship	○	○	○	○	○
	1 most important	2	3	4	5 least important

Workplace training, including strategies for new staff, such as providing an integrated care manual and shadowing opportunities for the new staff member to be placed with different professionals across sectors and services	◯	◯	◯	◯	◯
	1 most important	2	3	4	5 least important
Workplace training, including team meetings, mutual education about workflow or processes or a review of a problematic shared case	◯	◯	◯	◯	◯
	1 most important	2	3	4	5 least important
Short courses, such as motivational interviewing	◯	◯	◯	◯	◯
	1 most important	2	3	4	5 least important
Understanding of primary care providers, including how to interface and refer clients	◯	◯	◯	◯	◯
	1 most important	2	3	4	5 least important
Interprofessional skill development for faculty and a willingness and ability for faculty to evaluate and update curriculum in line with changes within the healthcare environment	◯	◯	◯	◯	◯
	1 most important	2	3	4	5 least important
Blended learning approaches that use discussions among participants, role play, problem-based learning and case application	◯	◯	◯	◯	◯
	1 most important	2	3	4	5 least important

Provide opportunities for students and healthcare workers to develop interpersonal strategies to consult, coordinate and collaborate routinely in practice	◯	◯	◯	◯	◯
	1 most important	2	3	4	5 least important
Create opportunities and a focus on building relationships and care pathways with organisations in the community	◯	◯	◯	◯	◯
	1 most important	2	3	4	5 least important
Include opportunities for critical thinking and reflective practice and the use of case presentations and role-plays	◯	◯	◯	◯	◯
	1 most important	2	3	4	5 least important
Create opportunities for all disciplines to train, think, create and seek solutions as a unit	◯	◯	◯	◯	◯
	1 most important	2	3	4	5 least important
Create an environment where there is a willingness to think differently about how services are delivered to meet the changing needs and expectations of people using health and social care services	◯	◯	◯	◯	◯
	1 most important	2	3	4	5 least important
Opportunities for broader and more meaningful engagement across health and social care	◯	◯	◯	◯	◯
	1 most important	2	3	4	5 least important
Incorporate and encourage innovative training and development that spans across health and social care	◯	◯	◯	◯	◯

	1 most important	2	3	4	5 least important
Design clinical practice environments to support and enable continuous learning that benefits not just learners, but also patients, communities and providers	◯	◯	◯	◯	◯

	1 most important	2	3	4	5 least important
Provide opportunities for participants to gain placement experience engaging in team-based assessments and intervention strategies	◯	◯	◯	◯	◯

Can you suggest any other models of training that may be missing from this list?

____

Would you like to participate in a semi structured interview for this study?

◯ Yes
◯ No

Can you suggest any other organisations who could be contacted to participate in this study?

____

Would you like a summary of the results of the research findings? This will be in the form of a brief lay summary.

◯ Yes
◯ No

As this is a Delphi study we will need to email you further questionnaires, can you please include your best email contact for this

____
