# Supplementary material for: An International Competency Framework for High-Quality Workforce Development in Integrated Care (IC): A Modified Delphi Study Among Global Participants
Source: Int J Integr Care. 2024 Apr 29;24(2):11. doi: 10.5334/ijic.8258 (PMC11067980; doi:10.5334/ijic.8258)
Supplement: Appendix B. — Delphi study survey Round 1: Results. [file ijic-24-2-8258-s2.pdf]

Appendix B (Supplementary files): Delphi study survey Round 1: Results

Tables B1 and B2 show the results of Round 1. Panellists were asked to rate the importance of 33 competencies and 17 themes on a 5-point scale, with 5 being least important and 1 being the most important.

Table B1 shows in order the highest-ranked competencies based on the number and percentage of panellists who rated each competency as "most important" (1). The last column shows the average importance rating for each competency (1–5). The lower the average, the higher its perceived importance by the expert group. A note to the table shows additional competencies and themes added by panellists.

Table B1. The number and percentage of experts (n = 21) who rated each competency as "most important" and the average score of importance per competency.

Competency	Rank	Number of panellists (n = 21) who rated this competency as "most important"	Percentage (%)	Average importance ranging from (1–5)[1]
Working effectively as a member of an interprofessional team	1	18	86%	1.2
Focusing on the needs of individuals, families, and communities to improve their quality of care, health outcomes and well-being	2	17	81%	1.3
Collaborating with other providers; strong communication and collaboration skills and the ability to develop strong working relationships with team members are imperative	2	17	81%	1.4
Patient-centred and relationship-centred care	3	16	76%	1.5

Competency	Rank	Number of panellists (n = 21) who rated this competency as "most important"	Percentage (%)	Average importance ranging from (1–5)[1]
A holistic understanding of individuals' health and well-being, capabilities, self-management abilities, needs, preferences and the environment in which they find themselves, including recognition that an individual's situation is dynamic, not static and requires regular monitoring	3	16	76%	1.3
Embracing individuals, communities, and services as partners in care	5	14	66%	1.3
A person-focused approach that considers the patient's presenting problem and other medical issues	5	14	66%	1.5
Patient-centredness; understanding and facilitating patients' pathways through the care system	5	14	66%	1.5
Involvement of and communication with caregivers	6	13	62%	1.5
Supporting patients in their involvement in their care by empowering them with knowledge and skills per their capabilities	6	13	62%	1.6
Skills to collaborate with community-based partners to improve patient care, including services outside traditional health related settings	7	12	57%	1.6
Familiarity with local and national resources to support social needs and connecting patients and caregivers to such resources, including community-based partners	8	10	48%	1.8
Collaborating with individuals and families to develop a personalised care plan to promote health and well-being that incorporates integrative approaches, including lifestyle counselling and mind-body strategies	8	10	48%	1.9

Competency	Rank	Number of panellists (n = 21) who rated this competency as "most important"	Percentage (%)	Average importance ranging from (1–5)[1]
An active approach to caregiver wellness and support, including understanding risk factors, recognising signs of caregiver distress, assessing caregiver needs and referring caregivers to care	9	9	43%	1.7
Enhancing workforce understanding of and exposure to alignment of activities across both the health and social care systems	10	8	38%	1.7
Enabling workforce attitudes to proactively pursue depth to understand system complexity, including knowledge of as well as how to access and refer to services	11	7	33%	1.9
Facilitating behaviour change in individuals, families, and communities to achieve ways of living that promote health, resilience, well-being, and disease prevention	11	7	33%	2.0
Valuing continuous learning, becoming mentors, teachers, and peer learners	11	7	33%	1.9
Consideration for concerns specific to vulnerable populations and their needs	12	6	28%	2.0
Skills to establish a longitudinal alliance with patients and their families	12	6	28%	2.0
Demonstrating skills to incorporate integrative health care into community settings and the healthcare system at large.	12	6	28%	2.4
Extensive integrated knowledge of biopsychosocial aspects of disease, systems of care and social determinants of care	14	4	19%	2.4
Understanding how to apply knowledge of the major determinants of health given resources available, relevant health policies and system design within a community	14	4	19%	2.4

Competency	Rank	Number of panellists (n = 21) who rated this competency as "most important"	Percentage (%)	Average importance ranging from (1–5)[1]
Obtaining an integrative health history that includes mind-body-spirit, nutrition, and use of both conventional and integrative therapies	14	4	19%	2.7
Community-based health education, health promotion and disease prevention	14	4	19%	2.3
Demonstrating basic knowledge of the major health professions, both integrative and conventional	15	3	14%	2.5
Practising self-care	15	3	14%	2.4

[1] 1 being "most important" and 5 being "least important".

Note. Additional competencies focused on the care coordination role, being able to challenge the practice of others, digital literacy, and practitioner attributes such as empathy and emotional intelligence, reflective thinking and learning and explicit values such as vision building in teams, systems thinking and leadership.

Table B2 shows in order the highest-ranked themes based on the number and percentage of panellists who rated each theme as "most important" (1). The last column shows the average rating of importance for each theme (1–5). The lower the average, the higher its perceived importance by the expert group. A note to the table shows additional themes added by participants.

Table B2. The number and percentage of experts (n = 21) who rated each theme as "most important" and the average score of importance per theme.

Themes	Rank	Number of panellists (n = 21) who provided this ranking for this theme	Percentage (%)	Average score of importance (1–5)[1]
Interprofessional teamwork and collaborative practice	1	18	86%	1.2
The development of skills to support person-centred care	2	16	76%	1.3
Empowering patients and communities to stay well	3	15	71%	1.4
Improved patient experience of health care	4	13	62%	1.6
Caregiver involvement	5	12	57%	1.5

Themes	Rank	Number of panellists (n = 21) who provided this ranking for this theme	Percentage (%)	Average score of importance (1–5)[1]
Implementing shared learning and innovation to encourage reform thinking and new ways of working	6	11	52%	1.7
Clinician/consumer collaboration: Adopting collaborative practices with consumers, carers and families and service providers working together	6	11	52%	1.7
Developing leaders, role models and local champions to support and implement reform and change	6	11	52%	1.6
Deeper understanding of our patients	7	10	47%	1.9
Deeper understanding of our communities	8	9	43%	1.8
Deeper understanding of our health and social care systems	9	8	38%	1.8
Improved services matched to community need	9	8	38%	1.8
Enhanced understanding of systems and available resources	10	7	33%	1.8
Illness prevention	10	7	33%	2.1
Skills to support people and communities to manage health and wellness	10	7	33%	2.0
Health promotion and disease prevention	11	6	28%	2.0
Improved quality of life for health and social care providers	11	6	28%	1.8

[1] 1 being the “most important” and 5 being the “least important”.

Note. Additional themes suggested an emphasis on the care coordination role and building people’s collaborative capacity as well as the importance of being able to share data across service providers.
