# Supplementary material for: An International Competency Framework for High-Quality Workforce Development in Integrated Care (IC): A Modified Delphi Study Among Global Participants
Source: Int J Integr Care. 2024 Apr 29;24(2):11. doi: 10.5334/ijic.8258 (PMC11067980; doi:10.5334/ijic.8258)
Supplement: Appendix C. — Delphi study Round 2 revised framework (Framework A). [file ijic-24-2-8258-s3.pdf]

Appendix C (Supplementary files): Delphi study Round 2 revised framework (Framework A)

Look overall at the themes and related competencies below.

Using the REDCap survey, comment if any should be removed or extra competencies included.

Suggest any rewording or how the framework could be improved.

Please keep in mind that these themes and competencies are specific to providing integrated care.

(0.1) Theme 1: Interprofessional teamwork and collaborative practice

1. Working effectively as a team member across a range of disciplines and service providers within the acute, primary, and social care settings (transdisciplinary teams).
2. Strong communication skills and the ability to develop robust working relationships within and across these settings.
3. Collaborating with a range of health and social care providers including community-based partners ensuring strong communication and collaboration skills.
4. Communicating and sharing information and data across teams and service providers and with patients and their families including digital platforms and social media.
5. The ability to build others' collaborative capacity.

(0.2) Theme 2: Care coordination role

6. The ability to adopt a care coordination and case management role, including effective communication with people/carers as well as service providers to improve the patient experience of health care.
7. Well-developed negotiation skills within teams, across services and with others in the network of care.
8. The ability to work within a complex system, collaborating with and within networks.
9. Understanding service provider roles including local and national strategies and programs and the ability to articulate these roles and programs to others.

(0.3) Theme 3: Person-centred care

10. Constructing a comprehensive understanding of the whole person's perspective, including their health needs, goals and how these can be met within their surrounding health and social care environment.
11. Focusing not only the needs of individuals but including families and communities to improve quality of care, health outcomes and well-being.
12. Having a comprehensive understanding of consumers' needs, including their health literacy, individual goals and how these can be met within their surrounding health and social care environments.
13. A holistic understanding of individuals' health and well-being, capabilities, self-management abilities, needs, preferences and the environment in which they find themselves, including recognition that an individual's situation is dynamic, not static, and requires regular monitoring.
14. Recognising consumers' health and well-being capabilities, self-management skills, support structures, needs and preferences and their environment and the system within which they interact.
15. Improving patients' and carers' knowledge and confidence in navigating the local health and social care system.
16. Supporting patients in their involvement in their care by empowering them with knowledge and skills per their capabilities.
17. Involvement of and communication with caregivers. This includes an active approach to caregiver wellness, ensuring that the carer understands the patient's risk factors and treatment plan, recognising signs of caregiver distress, assessing caregiver needs and referring caregivers to care and services to support them.

(0.4) Theme 4: Leadership

18. Developing leaders, role models and local champions to support and implement reform and change.
19. Demonstrating leadership in influencing other professionals and service providers to be more person-centred and collaborative in their practice.
20. Implementing opportunities for shared learning and innovation to encourage reform and new ways of working.
21. The ability to constructively challenge the practice of others.

22. Understanding governance and the benefits of information sharing between multi-stakeholders and sectors.
23. Explicit values and vision building in teams/systems thinking leadership change methods.
24. Using evaluation and research to drive change and improve services.

(0.5) Theme 5: Health promotion and disease prevention

25. Facilitating behaviour change in individuals, families and communities to achieve ways of living that promote health, resilience, well-being and disease prevention.
26. Obtaining an integrative health history that includes wellness strategies, nutrition and use of both conventional and integrative therapies.
27. Knowledge of and referral to preventative facilities and local programs to support lifestyle interventions.
28. Knowledge of how to teach self-care strategies to stay healthy and how to incorporate consumers' strengths and resources within their care plan.

(0.6) Theme 6: Population health approach

29. Understanding our communities where we work, including population needs and available resources.
30. Knowledge of local and national strategies and programs to support health and maintain wellness, including knowing when and how to access these services.
31. Understanding and navigating system complexity across programs and service providers.
32. Consideration for support and access to services for vulnerable populations.
33. An understanding of how social and cultural factors affect health.
34. Embracing individuals, communities and services as partners in care.

(0.7) Theme 7: Practitioner assets

Members of our health and social care workforce need to be seen as role models. They need to:

35. practise and integrate self-care strategies.
36. value continuous learning and maintain evidence-informed practice.
37. become mentors, teachers and peer learners.
38. show empathy and emotional intelligence.

39. practise reflective thinking and learning.
40. have digital literacy and knowledge of complementary and augmented technologies.

(0.8) Models of training

1. Include people with lived experience in all aspects of education and training, including design, delivery and evaluation.
2. Incorporate integrated care concepts throughout the curriculum of healthcare providers so they become fundamental to delivering care.
3. Focus on the inclusion of interprofessional training.
4. Provide opportunities for participants to gain placement experience engaging in team-based assessments and intervention strategies.
5. Design clinical practice environments to support and enable continuous learning that benefits not just learners but also patients, communities and providers.
6. Incorporate and encourage innovative training and development that spans health and social care.
7. Create an environment where there is a willingness to think differently about how services are delivered to meet the changing needs and expectations of people using health and social care services.
8. Provide opportunities for broader and more meaningful engagement across health and social care.
9. Create opportunities for all disciplines to train, think, create and seek solutions as a unit.
10. Include opportunities for critical thinking and reflective practice and the use of case presentations and role-play.
11. Create opportunities and a focus on building relationships and care pathways with organisations in the community.
12. Provide opportunities for students and healthcare workers to develop interpersonal strategies to consult, coordinate and collaborate routinely in practice.
13. Apply blended learning approaches that use discussions among participants, role-play, problem-based learning and case application.
14. Provide interprofessional skill development for faculty who are willing and able to evaluate and update curriculum in line with changes within the healthcare environment.

15. Offer understanding of primary care providers, including how to interface with and refer clients.
16. Provide short courses, such as motivational interviewing.
17. Offer workplace training, including team meetings, mutual education about workflow processes or a review of a problematic shared case.
18. Offer workplace training, including strategies for new staff, such as providing an integrated care manual and shadowing opportunities that place new staff members with different professionals across sectors and services.
19. Provide mentorship.
20. Provide trainees with education around maintaining wellness and work–life balance.
21. Embed structures to support collaboration and learning across services, strengthening multi-sector relationships and multi-organisation training.
22. Incorporate simulation-based scenarios using actors from the local community with lived experiences.
23. Incorporate the need to and how to provide education and support for caregivers, including illness prevention and improving quality of life.
24. Allow more time for networking and opportunities for individual service presentations and diverse attendance, including the social care and voluntary sectors.
25. Encourage case studies, exercises and simulations to allow students to interact with the content in as realistic a venue as possible.
26. Focus on soft skills, such as communication, teamwork and relationship building.
27. Focus on self-management promotion and skills, including the use of motivational interviewing techniques.
28. Focus on skills to build durable relationships with patients, other professionals, other services and caregivers.
29. Provide skills to navigate the health and social care systems and work on individualised care plans and assessments.
