# Supplementary material for: An International Competency Framework for High-Quality Workforce Development in Integrated Care (IC): A Modified Delphi Study Among Global Participants
Source: Int J Integr Care. 2024 Apr 29;24(2):11. doi: 10.5334/ijic.8258 (PMC11067980; doi:10.5334/ijic.8258)
Supplement: Appendix D. — Delphi study survey Round 2. [file ijic-24-2-8258-s4.pdf]

Round 2

Please complete the survey below.

Thank you!

Dear participants, thank you for completing Round 1 of the Delphi survey on workforce development in integrated care. I have attached the results of the survey showing how you rated the competencies and themes.

[Attachment: "Delphi Study Round 1 Results.pdf"]

Please download a copy of the participant Information Sheet for your records

______

[Attachment: "USYD Participant Information Sheet (Delphi Study - Round 2) .pdf"]

As part of Delphi study 2 - I have attached a 2 page document that shows an analyzed summary of the results. This summary (framework A) includes 7 domains (themes) and a list of revised competencies under each domain. You will now be asked a few simple questions as to (1) whether or not each domain should be kept, deleted, changed or rephrased and (2) whether or not each of the competencies under each domain should be kept, deleted, changed or rephrased.

[Attachment: "Delphi Study 2 Revised Framework Final.pdf"]

Before you start can you please indicate how many years experience you have in delivering or designing education and training that is specific to integrated care?

____
(whole years)

Looking at the 7 main domains (themes) of an international competency framework (Framework A) with the aim of building a global integrated care professional workforce, please rate whether each domain should be kept, deleted or changed.

Domain 1 - Skills to demonstrate Interprofessional Teamwork and Collaborative Practice

- ◯ Keep
- ◯ Delete
- ◯ Rephrase/change

Please provide suggested changes

____

Domain 2 - Skills to demonstrate Care Coordination

- ◯ Keep
- ◯ Delete
- ◯ Rephrase/change

Please provide suggested changes

____

Domain 3 - Skills to demonstrate Person Centred Care

- ◯ Keep
- ◯ Delete
- ◯ Rephrase/change

Please provide suggested changes

____

Domain 4 - Skills to demonstrate Leadership	◯ Keep<br>◯ Delete<br>◯ Rephrase/change
Please provide suggested changes	__________________________________
Domain 5 - Skills to implement Health Promotion and Disease Prevention	◯ Keep<br>◯ Delete<br>◯ Rephrase/change
Please provide suggested changes	__________________________________
Domain 6 - Knowledge to apply a comprehensive Population Health Approach to care	◯ Keep<br>◯ Delete<br>◯ Rephrase/change
Please provide suggested changes	__________________________________
Domain 7 - Practitioner Attributes (Personal values and attitudes)	◯ Keep<br>◯ Delete<br>◯ Rephrase/change
Please provide suggested changes	__________________________________

I have now listed the competencies under each of the domains can you please choose if you would like to keep, delete or rephrase/change each competency.

Domain 1 - Interprofessional Teamwork and Collaborative Practice.

1. Work effectively as a team member across a range of disciplines and service providers within the acute, primary, and social care settings and beyond (Transdisciplinary teams).

◯ Keep
◯ Delete
◯ Rephrase/change

Please provide suggested changes

____

2. Develop robust working relationships within and across these settings.

◯ Keep
◯ Delete
◯ Rephrase/change

Please provide suggested changes

____

3. Collaborate with a range of health and social care providers including community-based partners

◯ Keep
◯ Delete
◯ Rephrase/change

Please provide suggested changes

____

4. Share information and data across teams and service providers and with individuals and their families including digital platforms and social media

◯ Keep
◯ Delete
◯ Rephrase/change

Please provide suggested changes

____

5. Build others collaborative capacity.

◯ Keep
◯ Delete
◯ Rephrase/change

Please provide suggested changes

____

Domain 2 - Care Coordination Role

6. Adopt a care coordination and case management role, including effective communication with people/carers as well as service providers to improve the experience of health care	◯ Keep<br>◯ Delete<br>◯ Rephrase/change
Please provide suggested changes	__________________________________
7. Well-developed negotiation skills within teams, across services and with others in the network of care.	◯ Keep<br>◯ Delete<br>◯ Rephrase/change
Please provide suggested changes	__________________________________
8. The ability to coordinate care within a complex system	◯ Keep<br>◯ Delete<br>◯ Rephrase/change
Please provide suggested changes	__________________________________
9. Demonstrate knowledge of service providers including local and national strategies and programs and the ability to articulate the roles of these programs to others.	◯ Keep<br>◯ Delete<br>◯ Rephrase/change
Please provide suggested changes	__________________________________
10. The capacity to identify, which professionals and partners need to be included in the collaboration, based on the needs of the individual, population or community needs. Including the ability to invite and work closely with these service providers across a range of settings.	◯ Keep<br>◯ Delete<br>◯ Rephrase/change
Please provide suggested changes	__________________________________
11. The ability to use a range of technology to support care coordination.	◯ Keep<br>◯ Delete<br>◯ Rephrase/change
Please provide suggested changes	__________________________________

Domain 3 - Person Centred Care

12. Include families and communities when identifying the needs of individuals

○ Keep
○ Delete
○ Rephrase/change

Please provide suggested changes

____

13. Develop a comprehensive understanding of individuals needs including their health literacy, individual goals and how these can be met within their surrounding health and social care environment

○ Keep
○ Delete
○ Rephrase/change

Please provide suggested changes

____

14. Improve individuals' and carers' knowledge, skills and confidence in navigating the local health and social care system

○ Keep
○ Delete
○ Rephrase/change

Please provide suggested changes

____

15. Empower people to be able to take part in their own care, building on their capabilities

○ Keep
○ Delete
○ Rephrase/change

Please provide suggested changes

____

16. Involvement of and communication with caregivers. This includes an active approach to caregiver wellness, ensuring that the carer understands the person's risk factors and treatment plan, recognizing signs of caregiver distress, assessing caregiver needs and referring caregivers to care and services to support them.

○ Keep
○ Delete
○ Rephrase/change

Please provide suggested changes

____

Domain 4 - Leadership

17. Develop as leaders, role models and local champions to support and implement reform and change in integrated care	◯ Keep<br>◯ Delete<br>◯ Rephrase/change
Please provide suggested changes	____________________
18. Demonstrate leadership in influencing other professionals and service providers to be more person centred and collaborative in their practice	◯ Keep<br>◯ Delete<br>◯ Rephrase/change
Please provide suggested changes	____________________
19. Implement opportunities for shared learning and innovation across disciplines and industries to encourage reform and new ways of working	◯ Keep<br>◯ Delete<br>◯ Rephrase/change
Please provide suggested changes	____________________
20. The ability to constructively challenge the practice of others	◯ Keep<br>◯ Delete<br>◯ Rephrase/change
Please provide suggested changes	____________________
21. Demonstrate the benefits of shared governance between multistakeholders and sectors	◯ Keep<br>◯ Delete<br>◯ Rephrase/change
Please provide suggested changes	____________________
22. The ability to enable and create opportunities for systems thinking and change	◯ Keep<br>◯ Delete<br>◯ Rephrase/change
Please provide suggested changes	____________________
23. Use evaluation and research to drive change and improve services	◯ Keep<br>◯ Delete<br>◯ Rephrase/change
Please provide suggested changes	____________________

Domain 5 -Health Promotion and Disease Prevention

24. Facilitate behavior change in individuals, families, and communities to achieve ways of living that promote health, resilience, wellbeing, and disease prevention

◯ Keep
◯ Delete
◯ Rephrase/change

Please provide suggested changes

____

25. Obtain an integrative health history that includes wellness strategies, nutrition, and use of both conventional and integrative therapies

◯ Keep
◯ Delete
◯ Rephrase/change

Please provide suggested changes

____

26. Knowledge of and referral to preventative facilities and local programmes to support lifestyle interventions

◯ Keep
◯ Delete
◯ Rephrase/change

Please provide suggested changes

____

27. Demonstrate how to teach self-care strategies to stay healthy and how to incorporate individual's strengths and resources within their care plan

◯ Keep
◯ Delete
◯ Rephrase/change

Please provide suggested changes

____

Domain 6 -Population Health Approach

28. Identify and recognize the needs of local communities including population and available resources	◯ Keep<br>◯ Delete<br>◯ Rephrase/change
Please provide suggested changes	__________________
29. Knowledge of local and national strategies and programs to support health and maintain wellness including knowing when and how to access these services.	◯ Keep<br>◯ Delete<br>◯ Rephrase/change
Please provide suggested changes	__________________
30. Understand and navigate system complexity across programs and service providers	◯ Keep<br>◯ Delete<br>◯ Rephrase/change
Please provide suggested changes	__________________
31. Provide access and refer to programs to support vulnerable populations	◯ Keep<br>◯ Delete<br>◯ Rephrase/change
Please provide suggested changes	__________________
32. An understanding of how social and cultural factors affect health	◯ Keep<br>◯ Delete<br>◯ Rephrase/change
Please provide suggested changes	__________________

Domain 7- Mission Statement - Practitioner Attributes (Personal values and attitudes)

33. Practice and integrate self-care strategies.
◯ Keep
◯ Delete
◯ Rephrase/change

Please provide suggested changes

____

34. Value continuous learning and maintain evidence-informed practice
◯ Keep
◯ Delete
◯ Rephrase/change

Please provide suggested changes

____

35. Become mentors, teachers, and peer learners
◯ Keep
◯ Delete
◯ Rephrase/change

Please provide suggested changes

____

36. Show empathy and emotional intelligence
◯ Keep
◯ Delete
◯ Rephrase/change

Please provide suggested changes

____

37. Practice reflective thinking and learning
◯ Keep
◯ Delete
◯ Rephrase/change

Please provide suggested changes

____

38. Demonstrate digital literacy across a broad range of settings
◯ Keep
◯ Delete
◯ Rephrase/change

Please provide suggested changes

____
